# Molecular survey of *Babesia* parasites in Kenya: first detailed report on occurrence of *Babesia bovis* in cattle

**DOI:** 10.1186/s13071-022-05279-7

**Published:** 2022-05-07

**Authors:** Naftaly W. Githaka, Richard P. Bishop, Jan Šlapeta, David Emery, Edward K. Nguu, Esther G. Kanduma

**Affiliations:** 1 International Livestock Research Institute (ILRI), Nairobi, Kenya; 2 Washington State University, Pullman, WA USA; 3 Sydney School of Veterinary Science, Faculty of Science, The University of Sydney, Sydney, NSW 2006 Australia; 4 Department of Biochemistry, Faculty of Science and Technology, University of Nairobi, Nairobi, Kenya

**Keywords:** Babesiosis, *Rhipicephalus microplus*, Tick-borne diseases, Transboundary diseases, Ticks, *Babesia bovis*, *Babesia bigemina*, Real-time PCR

## Abstract

**Background:**

Among protozoan parasites in the genus *Babesia*, *Babesia bigemina* is endemic and widespread in the East African region while the status of the more pathogenic *Babesia bovis* remains unclear despite the presence of the tick vector, *Rhipicephalus microplus*, which transmits both species. Recent studies have confirmed the occurrence of *R. microplus* in coastal Kenya, and although *B. bovis* DNA has previously been detected in cattle blood in Kenya, no surveillance has been done to establish its prevalence. This study therefore investigated the occurrence of *B. bovis* in cattle in Kwale County, Kenya, where *R. microplus* is present in large numbers.

**Methods:**

A species-specific multiplex TaqMan real-time PCR assay targeting two *Babesia bovis* genes, 18S ribosomal RNA and mitochondrially-encoded cytochrome *b* and *B. bigemina* cytochrome *b* gene was used to screen 506 cattle blood DNA samples collected from Kwale County for presence of *Babesia* parasite DNA. A sub-set of 29 *B. bovis* real-time PCR-positive samples were further amplified using a *B. bovis*-specific spherical body protein-4 (SBP-4) nested PCR and the resulting products sequenced to confirm the presence of *B. bovis*.

**Results:**

A total of 131 animals (25.8%) were found to have bovine babesiosis based on real-time PCR. Twenty-four SBP4 nucleotide sequences obtained matched to *B. bovis* with a similarity of 97–100%. Of 131 infected animals, 87 (17.2%) were positive for *B. bovis* while 70 (13.8%) had *B. bigemina* and 26 (5.1%) were observed to be co-infected with both *Babesia* species. A total of 61 animals (12.1%) were found to be infected with *B. bovis* parasites only, while 44 animals (8.7%) had *B. bigemina* only. *Babesia bovis* and *B. bigemina* infections were detected in the three Kwale sub-counties.

**Conclusion:**

These findings reveal high prevalence of pathogenic *B. bovis* in a Kenyan area cutting across a busy transboundary livestock trade route with neighbouring Tanzania. The *Babesia* multiplex real-time PCR assay used in this study is specific and can detect and differentiate the two *Babesia* species and should be used for routine *B. bovis* surveillance to monitor the spread and establishment of the pathogen in other African countries where *B. bigemina* is endemic. Moreover, these findings highlight the threat of fatal babesiosis caused by *B. bovis*, whose endemic status is yet to be established.

**Graphical Abtract:**

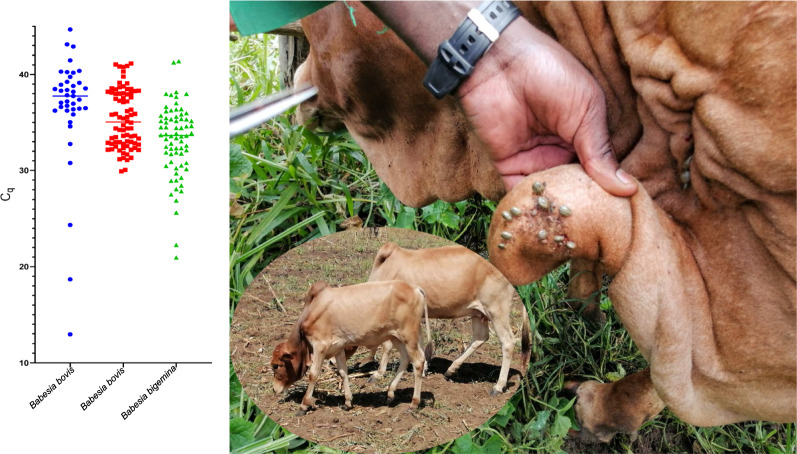

**Supplementary Information:**

The online version contains supplementary material available at 10.1186/s13071-022-05279-7.

## Background

Bovine babesiosis, the most economically important vector-borne disease of livestock globally, is mainly caused by protozoan parasites *Babesia bovis* and *Babesia bigemina* that are transmitted by tick species in the sub-genus *Boophilus* [1]. The disease causes major economic losses through animal mortality, poor growth rates, reduced milk yields in sick or recovered animals, and direct costs of tick control and disease treatment [2]. In Africa, *B. bigemina* is endemic and currently more widespread than *B. bovis*, reflecting the distribution of the indigenous tick vector *Rhipicephalus decoloratus*, which is more widely distributed in Africa, although *Rhipicephalus evertsi evertsi* is also a known vector [3]. *Rhipicephalus microplus*, which is a more competent vector of *B. bovis*, has become established in mainland Tanzania with evidence of displacement of the native *R. decoloratus* [4]. The tick had only been recorded in a small focus in coastal Kenya [5, 6]. However, in the past 15 years, *R. microplus,* which is a definitive vector for both *B. bovis* and *B. bigemina*, has been confirmed to occur in several countries in West Africa [7–9], Central Africa [10, 11] and recently East Africa [12–16]. The impact of this dispersal on the occurrence of *B. bovis* and the risk of pathogenic babesiosis in the affected African countries is yet to be assessed.

Clinical bovine babesiosis presents with significant haemolysis of the red blood cells, continuous fever, anaemia and often haemoglobinuria, which colours the urine reddish brown giving the disease the common name ‘red water’. Infections associated with *B. bovis* are often acute or subacute and have a shorter time course with more severe nervous symptoms rapidly leading to death or a protracted recovery rate in non-fatal cases [17]. In dairy cows, abortion and reduced or complete loss of milk (agalactia) are early signs of *Babesia* infection. Redwater causes high mortality and morbidity in susceptible livestock, especially in exotic and cross-breed cattle. Mortality rates of 30% for *B. bigemina* and 70–80% for the more pathogenic *B. bovis* infections have been observed [2]. Indigenous breeds of cattle can also be greatly affected by the less pathogenic *B. bigemina* under conditions of poor health or nutrition, a situation that is common in many vast areas of Africa, including Kenya [2], where other tick-borne diseases also occur. Babesiosis caused by *B. bigemina* is characterized by low parasitaemia of < 1%. In contrast, *B. bovis* infection has a high parasitaemia of > 10%, frequently with sequestration of infected red blood cells in cerebral capillaries resulting in symptoms which are often fatal. Cattle that recover from primary acute babesiosis, either naturally or after treatment, remain persistently infected and serve as a source of future tick infection and transmission [17, 18].

There is paucity of data on the status and occurrence of *R. microplus* outside the coastal counties in Kenya. Therefore, *R. decoloratus*, which is widely distributed in all agriculturally productive areas of eastern, central, Rift Valley and western Kenya [19], has been regarded as the major vector of bovine babesiosis in Kenya. Previously, McLeod and Kristjanson [20] predicted that 70% of Kenyan cattle were at risk from babesiosis and anaplasmosis with estimated annual economic losses amounting to $6.9 million per year. Co-infestation of animals with multiple tick species is typical, so co-infection with multiple tick-transmitted pathogens and significant disease burden are frequently encountered.

Infection with redwater in Kenya is mainly inferred from clinical signs; microscopic examination of blood smears for *Babesia* parasites is usually not performed because of the characteristic haematuria. Given the limited records of *R. microplus* in Kenya to date, redwater has always been attributed to the more ubiquitous *B. bigemina* vectored by *R. decoloratus* [21–26]. However, the recent confirmation of the occurrence of *R. microplus* in coastal Kenya by Kanduma et al. [13] indicates the existence of *B. bovis*. *Babesia bovis* DNA has been reported previously in cattle blood in Kenya [26, 27]. The use of molecular tests increases the sensitivity for detection and enables differentiation of *B. bovis* from other *Babesia* parasites. Kim et al. [28] and Zhang et al. [29] previously developed and validated highly sensitive quantitative qPCR TaqMan probes for detecting, quantifying and differentiating *B. bovis* from *B. bigemina*. In this study, we used these probes to investigate the occurrence of *B. bovis* in Kenya considering the recent reports of *R. microplus* in the local cattle populations.

## Methods

### Study site

**Fig. 1:**
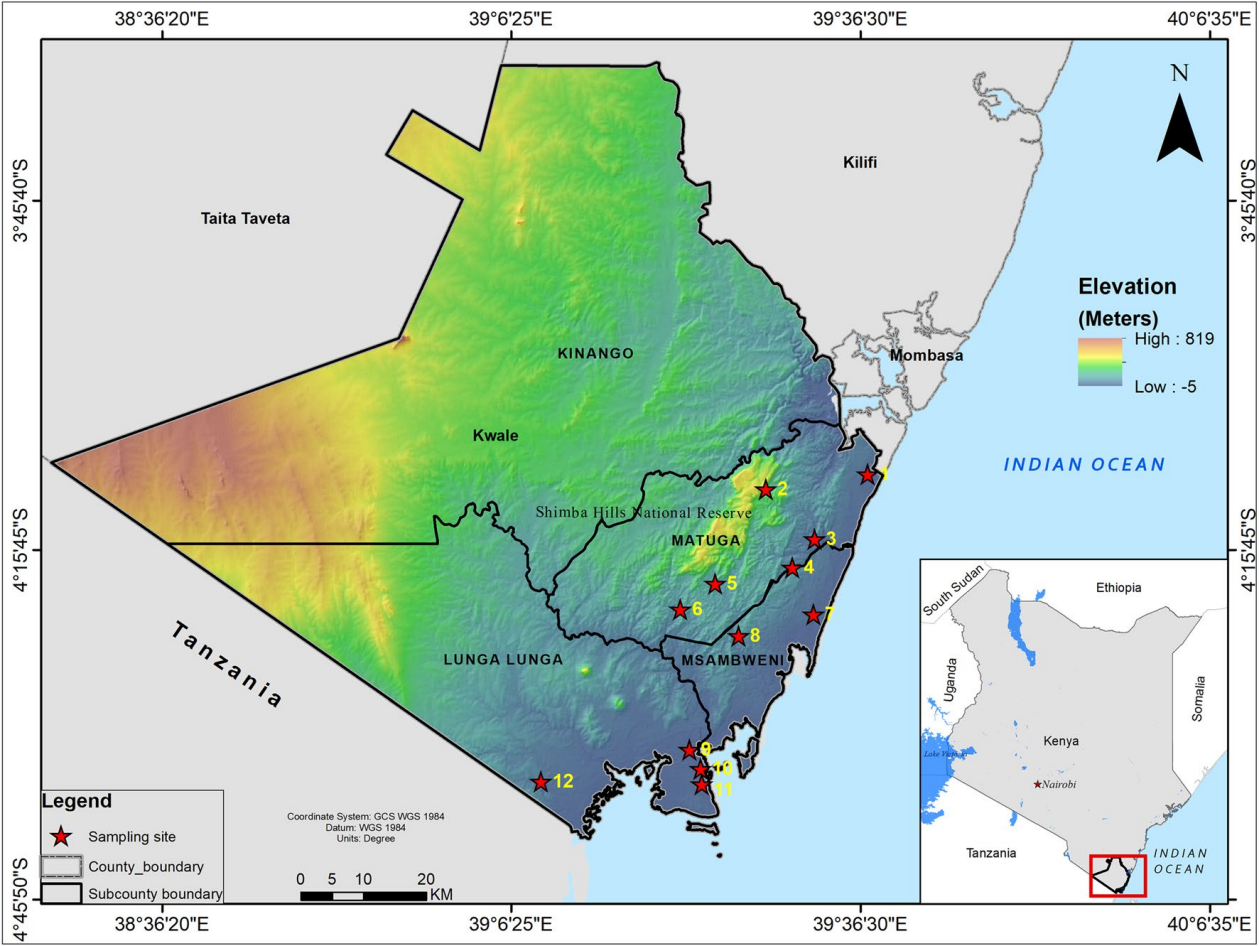
Map of Kwale County showing the 12 localities (shown in numbers) where the 506 cattle were sampled. The sampling localities were: (1) Miaji farm (*n* = 40), (2) Matuga (*n* = 13), (3) Ukunda (*n* = 28), (4) Mwanjaba (*n* = 10), (5) Kipabane (*n* = 43), (6) Kichaka simba (*n* = 107), (7) Majiboni (*n* = 32), (8) Tangini (*n* = 59), (9) Shimoni Kidimu (*n* = 34), (10) Shimoni (*n* = 32), (11) Kiwambale (*n* = 33) and (12) Vanga (*n* = 75). Topography data were acquired from ASTER Global Digital Elevation Model Version 3, while the shapefiles for administrative boundaries were obtained from the World Resource Institute website (https://www.wri.org). ArcGIS Desktop version 10.8 was used to prepare and analyse the layers and produce the final map

A cross-sectional baseline survey was conducted in May 2019 in 12 sites in Kwale County, Kenya (Fig. 1) to determine the occurrence of *B. bovis*. The county is situated along the Kenyan coastline neighbouring the Indian Ocean on the East and Southeast and Tanzania on the Southwest. The county has a tropical type of climate with an average temperature of 23 °C with a high of 25 °C in March and a low of 21 °C in July. Annual precipitation is < 800 mm with the coastal parts of the county receiving > 1000 mm of precipitation per year, while most of the central to west areas receive around 500–750 mm. Rainfall is bi-modal with a short rain season from October to December and a long rain season from April to July. Detailed geo-climatic characteristics of the county have previously been described [13].

### Cattle blood sampling

In total, 506 adult cattle were randomly sampled across 12 sites located in three sub-counties, namely, Matuga, Msambweni and Lunga Lunga (Fig. 1). Since this was a baseline survey to screen for presence of *Babesia* parasites, opportunistic sampling was done with no stratification at farm/household level and data on risk factors were also not collected. Five millilitres of blood was collected from the jugular vein into EDTA vacutainers (BD, USA). The samples were stored in a cool box with ice packs and later transported to the regional laboratory in Kwale where they were refrigerated at 4 °C for 3 days. The samples were later transported to the International Livestock Research Institute (ILRI) Laboratories in Nairobi where subsequent investigations were conducted.

### Whole-blood genomic DNA isolation

Whole cattle blood in EDTA tubes was thawed and thoroughly mixed by gentle rocking. Whole-blood genomic DNA was isolated from 300 µl of blood using the Promega Wizard^®^ genomic DNA purification kit (Promega Corporation, Madison, WI, USA) following the manufacturer’s protocol. DNA yield and purity were determined by spectrophotometry using Nanodrop2000 spectrophotometer (Thermo-Scientific, USA). The DNA was stored at − 20 °C until use.

### Detection of *Babesia* DNA

A TaqMan multiplex real-time PCR targeting two *Babesia bovis* and one *B. bigemina* genes was used to detect *Babesia* DNA. One *B. bovis* primer and probe set targeted the nuclear 18S rDNA [28] while the other targeted the mitochondrial cytochrome *b* gene [29]. The *B. bigemina* primers and probe were derived from the cytochrome *b* gene. Details of the primer and probe sequences used in the multiplex assay are listed in Additional file 1: Table S1. The real-time PCR was conducted in 200-µl 96-well plates in a QuantStudio™ 5 detection system (Thermo Fisher Scientific, Waltham, MA, USA). Samples were prepared in 20-µl reactions, which included 10 µl of 2 × Universal Probe qPCR Master Mix (New England Biolabs, Ipswich, MA, USA), 0.8 µl of 10 pmol of each oligonucleotide primer, 0.2 µl of 10 pmol of the fluorescence-labelled probe, 3 µl of 10 ng genomic DNA template and 5 µl of RNase/DNase free water. Temperature cycling parameters were 95 °C for 3 min followed by 45 cycles of 95 °C for 10 s and 54 °C for 25 s. Each PCR run included a *B. bovis* control sample extracted from an in vitro cell culture and *B. bigemina*-positive control sample obtained from an infected calf and a no-template PCR grade water sample as negative control. The cycle quantification (Cq) scores corresponding to the PCR cycle number at which the amplification curve of each sample intersected the threshold line were recorded for each sample.

### Standardization and construction of real-time PCR calibration curves

A standard curve was run singly for each of the primer and probe sets using tenfold serially diluted *B. bovis* control DNA sample (48 ng/µl) isolated from an in vitro culture and a *B. bigemina* positive control DNA sample (17 ng/µl) isolated from blood of an infected calf at the Tick Fever Centre (TFC), Queensland Department of Agriculture, Australia, ranging from 10^−1^ to 10^−8^. The diluted DNA was then amplified using a multiplexed assay of three primer and probe sets (*B. bovis* 18S, *B. bovis* cytochrome *b* and *B. bigemina* cytochrome *b*; Additional file 1: Table S1). Resulting data were analysed using the QuantStudio design and analysis software version 2.6.0. To generate the calibration curves, the cycle quantification (Cq) scores for the diluted samples were determined based on a preset baseline threshold of 0.074 ΔRn for *B. bovis* 18S and 0.02 ΔRn for *B. bovis* cytochrome *b* and *B. bigemina* cytochrome *b* (ΔRn is defined as the Rn value of an experimental reaction minus the Rn value of the baseline signal). The Cq scores were then plotted against corresponding DNA dilutions. The efficiency (*E*) of the primer sets expressed as a percentage of each of the individual real-time PCR assays was calculated from the slope of the respective standard curve. The coefficient of correlation (*R*^2^) of each standard curve was also determined using the QuantStudio analysis software.

### *Babesia bovis* spherical body protein-4 (SBP-4) nested PCR amplification and sequencing

A sub-set of 59 real-time PCR *B. bovis*-positive samples was subjected to standard PCR amplification and sequencing using specific primers targeting a 503 fragment of the highly conserved *B. bovis* spherical body protein-4 (SBP-4) in a nested PCR (nPCR) as previously described [30]. Primer sequences used in the nested PCR are listed in Additional file 1: Table S1. Initial PCR amplifications were done in a 25 μl-reaction mixture having 12.5 μl 2 × MyTaq™ Red Mix (Meridian Bioscience, USA) PCR buffer mix, 1 μl (10 pmol) of each primer, 3 μl DNA template and 7.5 μl nuclease free PCR water. The thermocycling conditions for the PCR amplifications were as follows: initial denaturation of 5 min at 95 °C followed by 35 cycles (1 min of denaturation at 94 °C, 1 min of annealing at 55 °C and 1 min of extension at 72 °C) and final extension at 72 °C in 10 min in a AllInOneCycler™ PCR system (Bioneer). A nested PCR was done using 2 μl of DNA template obtained from the primary PCR under the same amplification conditions. DNA control samples used in the real-time PCR were included as positive controls while nuclease free PCR water was used as a negative control. The nPCR products were purified and directly sequenced with both forward and reverse nested PCR primers at Macrogen Europe B.V (The Netherlands).

### Data analysis

Real-time PCR data were exported to Ms Excel where the mean and range of Cq scores was calculated. A box plot of Cq values detected was generated using STATA 15. The prevalence of each *Babesia* was estimated as the proportion of total samples for each gene and prevalence rates between sub-counties compared using chi-square. The *P* values for statistical significance were set at 0.05. To estimate the confidence intervals around the prevalence estimate, the function *prop.test* of the *stats package* implemented in R (R Core Team) was used, setting the confidence level at 95% to test the true proportion of the sample genes. The 95% confidence interval was estimated using the formula:

$$\overline{x } \pm z\alpha /2 \frac{\sigma }{\sqrt{n}}$$

where $\sigma$ is standard deviation, *n* is sample size, and $\overline{x }$ is proportion of estimate.

Spherical Body Protein-4 (SBP-4) amplicons were obtained with 29 of 59 field isolates amplified. Sequence chromatograms were obtained from 24 of the 29 field samples and three control samples sequenced. The chromatograms were visually inspected and resulting sequences edited manually using CLC Main Workbench 20 software (CLC bio, Qiagen GmbH, Germany). Five of the sequenced field samples returned low quality sequences and were omitted from further analysis. Obtained nucleotide sequences were trimmed to remove low-quality reads at the 5′ and 3′ ends and consensus sequences generated from both the assembled forward and reverse fragments. Molecular identity of the isolates was confirmed via Basic Local Alignment Search Tool for nucleotides (BLASTn) [31] searches of the SBP-4 sequences against the GenBank’s non-redundant nucleotide sequence database. The consensus nucleotide sequence from each isolate was then processed for further multiple sequence alignments using CLC Main Workbench 20 software (CLC bio, Qiagen GmbH, Germany). Percent identity analyses were performed using Clustal Omega multiple sequence analyses tool [32].

## Results

### Evaluation of *Babesia* real-time PCR assays: statistical parameters, standard curves and efficiency

**Fig. 2:**
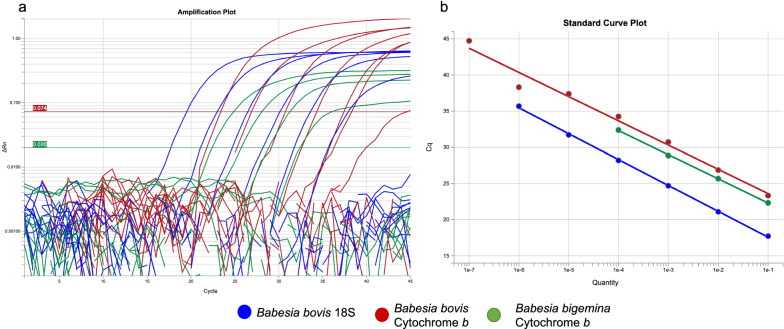
Real-time PCR amplification and calibration curves of the three primer and probe sets used in the detection of *Babesia* parasites in the current study. **a** Amplification plots. **b** Standard curves. The *Babesia bovis* 18S primer set had an efficiency of 98.96%, a slope of –3.34 and a correlation coefficient (*R*^2^) of 0.981, while the cytochrome *b* primers had an efficiency of 90.4%, a slope − 3.57 and a *R*^2^ of 0.999. The *B. bigemina* set had an efficiency of 99.51%, a slope of − 3.33 and a *R*^2^ of 1.0. The plots and standard curves were generated using QuantStudio design and analysis software version 2.6.0. The detection limit of the gene in the control DNA was 10^−7^ dilution for *B. bovis* 18S primers, 10^−6^ for cytochrome *b* and 10^−4^ for *B. bigemina* cytochrome *b*

Initially, control gDNA was used to evaluate the utility of a previously published set of real-time PCR primers targeting *B. bovis* and *B. bigemina* [28, 29]. A summary of the statistical parameters, standard curves and efficiencies of the primer sets used in the multiplex real-time PCR is shown in Table 1 and Fig. 2a, b. The lowest Cq value detected by the *B. bovis* 18S primer pair at the lowest serial dilution of 10^−1^ was 23.3 while the cytochrome *b* detected a Cq of 17.7. The *B. bigemina* cytochrome *b* primers detected a Cq of 22.3 at the lowest dilution of 10^−1^. The *B. bovis* 18S primers returned a maximum Cq value of 44.7 at 10^−7^ dilution, which was the detection limit of the control DNA, while the cytochrome *b* returned a high of 35.7 at a dilution of 10^−6^, which was the detection limit of the control DNA used. The *B. bigemina* cytochrome *b* primers returned a maximum of 32.4 at a dilution of 10^−4^, which was also the detection limit of the control DNA used (Table 1).

**Table 1:** Summary details of the statistics and efficiency parameters of the three real-time PCR primer sets

Sample name	Serial dilution level	DNA concentration	Gene	CT	*Y*-intercept	Slope	Efficiency
Bbov1	10^−1^	14.4 ng	*B. bovis* 18S	23.3	20.3	− 3.34	98.96
Bbov2	10^−2^	1.44 ng	*B. bovis* 18S	26.9	20.3	− 3.34	98.96
Bbov3	10^−3^	144 pg	*B. bovis* 18S	30.7	20.3	− 3.34	98.96
Bbov4	10^−4^	14.4 pg	*B. bovis* 18S	34.2	20.3	− 3.34	98.96
Bbov5	10^−5^	1.44 pg	*B. bovis* 18S	37.4	20.3	− 3.34	98.96
Bbov6	10^−6^	144 fg	*B. bovis* 18S	38.3	20.3	− 3.34	98.96
Bbov7	10^−7^	14.4 fg	*B. bovis* 18S	44.7	20.3	− 3.34	98.96
Bbov8	10^−8^	1.44 fg	*B. bovis* 18S	Undetermined	20.3	− 3.34	98.96
Bbov1	10^−1^	14.4 ng	*B. bovis* Cytochrome *b*	17.7	14	− 3.57	90.46
Bbov2	10^−2^	1.44 ng	*B. bovis* Cytochrome *b*	21.1	14	− 3.57	90.46
Bbov3	10^−3^	144 pg	*B. bovis* Cytochrome *b*	24.7	14	− 3.57	90.46
Bbov4	10^−4^	14.4 pg	*B. bovis* Cytochrome *b*	28.2	14	− 3.57	90.46
Bbov5	10^−5^	1.44 pg	*B. bovis* Cytochrome *b*	31.7	14	− 3.57	90.46
Bbov6	10^−6^	144 fg	*B. bovis* Cytochrome *b*	35. 7	14	− 3.57	90.46
Bbov7	10^−7^	14.4 fg	*B. bovis* Cytochrome *b*	Undetermined	14	− 3.57	90.46
Bbov8	10^−8^	1.44 fg	*B. bovis* Cytochrome *b*	Undetermined	14	− 3.57	90.46
Bbig1	10^−1^	51 ng	*B. bigemina* Cytochrome *b*	22.3	18.9	− 3.33	99.51
Bbig2	10^−2^	5.1 ng	*B. bigemina* Cytochrome *b*	25.7	18.9	− 3.33	99.51
Bbig3	10^−3^	510 pg	*B. bigemina* Cytochrome *b*	28.9	18.9	− 3.33	99.51
Bbig4	10^−4^	51 pg	*B. bigemina* Cytochrome *b*	32.4	18.9	− 3.33	99.51
Bbig5	10^−5^	5.1 pg	*B. bigemina* Cytochrome *b*	Undetermined	18.9	− 3.33	99.51
Bbig6	10^−6^	510 fg	*B. bigemina* Cytochrome *b*	Undetermined	18.9	− 3.33	99.51
Bbig7	10^−7^	51 fg	*B. bigemina* Cytochrome *b*	Undetermined	18.9	− 3.33	99.51
Bbig8	10^−8^	5.1 fg	*B. bigemina* Cytochrome *b*	Undetermined	18.9	− 3.33	99.51

A standard curve was run singly for each primer and probe set. The *B. bovis* 18S primer set had an efficiency of 98.96%. Its standard curve had a slope of -3.34 and a correlation coefficient (*R*^2^) of 0.981. The *Y*-Intercept was 20.2. The *B. bovis* cytochrome *b* primers had an efficiency of 90.4%, a slope of − 3.57, a *R*^2^ of 0.999 and a *Y*-intercept of 14.0. The *B. bigemina* set had an efficiency of 99.51%, slope of − 3.33, *R*^2^ of 1.0 and *Y*-intercept of 18.97. The amplification plots and standard curves of each primer set as determined from the Cq values of the serially diluted standard solutions are shown in Fig. 2a, b, respectively. The detection limit in the control DNA was 10^−7^ dilution for 18S gene, 10^−6^ dilution for *B. bovis* cytochrome *b* and 10^−4^ dilution for *B. bigemina* cytochrome *b*. The *B. bovis* 18S and cytochrome *b*, which were labelled with different fluorescence dyes, were not detected in the *B. bigemina* control, nor was *B. bigemina* cytochrome *b* detected in the *B. bovis* control included in each PCR run. Thus, the primer sets employed were species-specific for *B. bovis* and *B. bigemina* as no cross-reaction was observed with the respective controls. In all the PCR reactions carried out, a no-template water sample was included as a negative control to rule out false positives due to cross-contamination.

### Detection of *B. bovis* and *B. bigemina* DNA

**Fig. 3:**
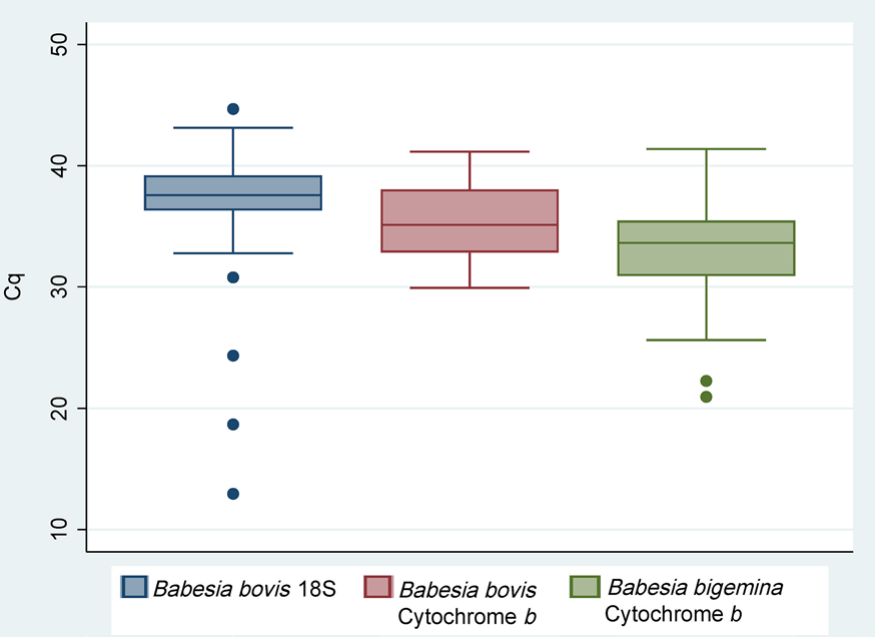
Box plot showing Cq score distribution range of the three real-time PCR target genes. The minimum Cq value was 12.9 for *Babesia bovis* 18S, 29.9 for *B. bovis* cytochrome *b* and 20.9 for *B. bigemina* cytochrome *b*. The median values were 37.5, 35.1 and 33.6 while maximum values were 44.7, 41.2 and 41.4 for *B. bovis* 18S, *B. bovis* cytochrome *b* and *B. bigemina* cytochrome *b*, respectively. The plot was generated using STATA 15

The real-time PCR primer sets employed in this study allowed for species-specific detection of *B. bovis* and *B. bigemina* DNA present in the screened blood samples. Since the efficiency parameters of the primers were within the recommended limits of a good quantitative PCR (qPCR) efficiency of between 90 and 110%, a slope that falls between − 3.6 and − 3.3 and a coefficient of correlation (*R*^2^) of > 0.98 (Table 1; Fig. 2b), all samples that returned Cq values of < 45 for the *B. bovis* 18S primers with a detectable amplification plot above the baseline threshold magnitude of 0.072 ΔRn were regarded as positives since the corresponding Cq values obtained with the cytochrome *b* primers for the same samples were lower (Additional file 2: Table S2). For the *B. bovis* and *B. bigemina* cytochrome *b* primer sets, all samples that returned Cq values of < 42 with a detectable amplification plot showing an exponential growth of the PCR product above the signal threshold of 0.02 ΔRn were also regarded as positives. A total of 131/506 samples returned Cq values that met these criteria and were thus classified as positive for either *B. bovis* or *B. bigemina*, or both. Detectable amplifications with the *B. bovis* 18S primers were observed in 39 samples, while a total of 87 samples resulted in detectable amplicons using the *B. bovis* cytochrome *b* primers. A list of the 131 field samples and their corresponding Cq values is shown in Additional file 2: Table S2. The range distribution of the Cq values detected by the three assays is shown in Fig. 3. The highest Cq value obtained with the field samples using the *B. bovis* 18S was 44.7 and the lowest was 12.9. The *B. bovis* cytochrome *b* returned a high Cq value of 41.2 and a low of 29.9 with a mean Cq value of 35.4 (SD ± 2.997). The *B. bigemina* cytochrome *b* primers returned a maximum of 41.4 and a minimum of 21. No detectable amplification was observed in 375 of the screened DNA samples.

Nucleotide BLAST analysis of 24 SBP-4 sequences obtained showed that they were homologous with *B. bovis* SBP-4. The percent sequence identities among isolates analysed in this study and top matching reference sequences from GenBank repository ranged between 97 and 100% (Table 2). They matched previously reported Kenyan isolates with a similarity of 96–100% [27]. Nine isolates shared 99–100% similarity with isolates from Benin reported by Moumouni et al. [33], while 13 isolates shared a similarity of 99.0–100% with South African isolates. Two isolates shared a 97.0% similarity with sequences from Indonesia reported by Guswanto et al. [34]. The 27 SBP-4 nucleotide sequences obtained were deposited in the GenBank database under the accession numbers ON012652-ON012678 (Table 2).

**Table 2:** Summary of BLASTn analysis hits showing the sequence accession numbers, top matching GenBank sequence, percent identity, animal host and country of origin for each of the 24 field and 3 control samples whose spherical body protein 4 gene was sequenced in the present study

Voucher number	Accession number	Matching organism	Percent identity (%)	Top matching sequence	Host	Country of origin	Reference
32	ON012652	*B. bovis*	100	KX685402.1	Cattle	Benin	[33]
95	ON012653	*B. bovis*	99	KF626634.1	Cattle	South Africa	Unpublished
96	ON012654	*B. bovis*	100	KX685402.1	Cattle	Benin	[33]
113	ON012655	*B. bovis*	100	KX685402.1	Cattle	Benin	[33]
341	ON012656	*B. bovis*	100	KF626637.1	Cattle	South Africa	Unpublished
357	ON012657	*B. bovis*	100	KF626637.1	Cattle	South Africa	Unpublished
380	ON012658	*B. bovis*	100	KX685402.1	Cattle	Benin	[33]
390	ON012659	*B. bovis*	97	KY484530.1	Cattle	Indonesia	[34]
419	ON012660	*B. bovis*	99	KX685402.1	Cattle	Benin	[33]
427	ON012661	*B. bovis*	100	KX685402.1	Cattle	Benin	[33]
430	ON012662	*B. bovis*	100	KF626637.1	Cattle	South Africa	Unpublished
440	ON012663	*B. bovis*	99	KF626632.1	Cattle	South Africa	Unpublished
451	ON012664	*B. bovis*	100	KX685402.1	Cattle	Benin	[33]
460	ON012665	*B. bovis*	100	KF626637.1	Cattle	South Africa	Unpublished
463	ON012666	*B. bovis*	100	KX685402.1	Cattle	Benin	[33]
468	ON012667	*B. bovis*	100	KF626637.1	Cattle	South Africa	Unpublished
470	ON012668	*B. bovis*	97	KY484530.1	Cattle	Indonesia	[34]
475	ON012669	*B. bovis*	99	KF626634.1	Cattle	South Africa	Unpublished
488	ON012670	*B. bovis*	99	KF626634.1	Cattle	South Africa	Unpublished
489	ON012671	*B. bovis*	100	KF626637.1	Cattle	South Africa	Unpublished
490	ON012672	*B. bovis*	100	KF626637.1	Cattle	South Africa	Unpublished
495	ON012673	*B. bovis*	100	KF626637.1	Cattle	South Africa	Unpublished
499	ON012674	*B. bovis*	99	KF626634.1	Cattle	South Africa	Unpublished
500	ON012675	*B. bovis*	100	KX685402.1	Cattle	Benin	[33]
Bbovis_Monash	ON012676	*B. bovis*	99	KY484534.1	Cattle	Indonesia	[34]
3347	ON012677	*B. bovis*	99	KY484534.1	Cattle	Indonesia	[34]
3377	ON012678	*B. bovis*	99	KY484534.1	Cattle	Indonesia	[34]

### Prevalence of *B. bovis* and *B*. *bigemina* infection

A total of 506 cattle blood samples were screened for presence of both *B. bovis* and *B. bigemina*. The distribution of the number of positive cases across the sampled sub-counties is shown in Table 3. The overall bovine babesiosis prevalence rate was 25.9% (131/506). Of the 131 *Babesia*-positive animals, 87 were found to be infected with *B. bovis*, indicating a prevalence of 17.2% (95% CI; 14.1–20.8%) (87/506), while 70 were positive for *B. bigemina*, giving a prevalence of 13.8% (70/506) (95% CI; 11.0–17.2%), and 27 (5.3%) (95% CI; 3.6–7.8%) had mixed infections of both *B. bovis* and *B. bigemina*. The 18S *B. bovis* primers detected 39 positive cases (7.7%) (95% CI; 5.6–10.4%), while the *B. bovis* cytochrome *b* primers detected 81 (16%) (95% CI: 12.9–19.6%) positive cases (Table 3). A total of 61 animals (12.1%) were found to have single *B. bovis* infection while 44 animals (8.7%) were positive for *B. bigemina* only.

**Table 3:** Distribution of *Babesia*-positive blood across the study sites

Sampling site	Total number of *Babesia*-positive samples by gene					
Sub-county	Number of animals	No. of animals *B. bovis* positive with 18S n (prevalence) (95% CI prevalence)	No. of animals *B. bovis* positive with cytochrome *b* *n* (prevalence) (95% CI prevalence)	No. of animals *B. bovis* positive with both 18S & cytochrome *b* genes n (prevalence) (95% CI prevalence)	No. of *B. bigemina* positive animals n (prevalence) (95% CI prevalence)	No. of animals having both *B. bovis* and *B. bigemina* mixed infection n (prevalence) (95% CI prevalence)
*Matuga*	203	6 (3.0%) (1.2–6.6%)	33 (16.3%) (11.6–22.2%)	34 (16.7%) (12.0–22.8%)	27 (13.3%) (9.1–18.9%)	9 (4.4%) (2.2–8.5%)
*Msambweni*	129	5 (3.9%) (1.4–9.3%)	6 (4.7%) (1.9–10.3%)	7 (5.4%) (2.4–11.3%)	13 (10.1%) (5.7–16.9%)	2 (1.6%) (0.3–6.1%)
*Lunga Lunga*	174	28 (16.1%) (11.1–22.6%)	42 (24.1%) (18.1–31.3%)	46 (26.4%) (20.2–33.8%)	30 (17.2%%) (12.1–23.9%)	16 (9.2%) (5.5–14.8%)
*Total*	506	39 (7.7%) (5.6–10.4%)	81 (16.0%) (12.9–19.6%)	87 (17.2%) (14.1–20.8%)	70 (13.8%) (11.0–17.2%)	27 (5.3%) (3.6–7.8%)

The prevalence of each *Babesia* infection was estimated as the proportion of total samples per sub-county with each gene. The sampling site (sub-county) was statistically associated with prevalence of *Babesia* infection (*χ*^2^ = 72.4, *df* = 8, *P* < 0.0001)

*Babesia bovis* infections were detected in all the three counties sampled. The highest *B. bovis* prevalence was observed in Lunga Lunga sub-county (52.9%; 46/87), while the lowest was observed in Msambweni (8%; 7/87). The highest prevalence of *B. bigemina* was also observed in Lunga Lunga (42.9%; 30/70), while the lowest was observed in Msambweni (18.6%; 13/70). Mixed infections were also highest in Lunga Lunga (59.3%; 16/27). The sampling site (sub-county) was statistically associated with prevalence of *Babesia* infection (χ^2^ = 72.4, *df* = 8, *P* < 0.0001).

## Discussion

Bovine babesiosis is one of the four major tick-borne diseases that affect livestock in Kenya [21–26]. Until recently, bovine babesiosis in the country was presumed to be caused by *B. bigemina*, transmitted principally by *R. decoloratus*, which is endemic in many regions in Kenya and tropical Africa [19]. Recent recordings of *R. microplus* in Kwale [13] indicated an urgent need to investigate the prevalence of pathogens transmitted by this tick including *B. bovis* to update epidemiological maps of these diseases. Using specific molecular assays, this study has confirmed the presence of pathogenic *B. bovis* DNA in cattle blood from coastal Kenya, with the findings representing the first report of a substantial level of occurrence of the parasite in Kenyan cattle.

Screening of the 506 cattle samples with two *B. bovis* and one *B. bigemina* specific probes, we observed that *B. bovis* was present in 17.2% (87/506) of the animals, while 13.8% (70/506) of the samples were positive for *B. bigemina*. Co-infections with the two *Babesia* spp. were observed in 27 (5.1%) of the animals screened (Table 3). This is also the first major study to report occurrence of the pathogenic *B. bovis* in the East African region in relatively high numbers. The findings indicate that *B. bovis* infections do occur in significant numbers in this region but are probably disregarded in most studies screening for tickborne pathogens in cattle because of the belief that only the endemic *B. bigemina* is present. This calls for implementation of surveillance and control measures because the economic burden of ticks and tick-borne diseases in Africa [2] and globally is very high [1].

In this study, prevalence of *Babesia* infection was significantly associated with sub-county (χ^2^ = 72.4, *df* = 8, *P* < 0.0001). The highest number of *B. bovis* infections was observed in Lunga Lunga sub-county, which borders Tanzania. There is a livestock market in Tanga, a port city in Northeast Tanzania, and at the Kenya border post at Lunga Lunga where cattle from Tanzania are purchased by Kenyans from neighbouring counties. Moreover, there are two holding sites, one at Lunga Lunga and the second at Msambweni, both in Kwale, where animals are held pending movement to final destinations of neighbouring counties and beyond. The Lunga Lunga market is frequented by Kenyan traders from as far as Tana River County while some are from Somalia. The extensive movement and transborder trade of potentially tick-infested and infected cattle is regarded as the major driving factor responsible for introduction of *Babesia*-infected tick vectors into new areas [17]. A similar pattern of transboundary cattle trade contributing to the spread of *R. microplus* and possibly its associated pathogens has been reported in Central and West Africa where animal movement across national boundaries is common [11]. High prevalence of the pathogenic *B. bovis* found in this study suggests fatal bovine babesiosis may become a severe threat to cattle across eastern African countries where the tick vector has been confirmed to be present. Our current report alongside recent confirmation of the occurrence of *R. microplus* in Kenya provides a rationale to implement surveillance to monitor potential disease outbreaks related to *B. bovis* infections. Effective measures should also be instituted to control the spread of *R. microplus* to limit transmission of *B. bovis* babesiosis especially further inward where both were previously absent.

The *B. bovis* real-time PCR and SBP-4 nested PCR tests used currently have added both reliability and sensitivity to the detection of *B. bovis* infections in Kenya. Classically, active *B. bovis* infection is diagnosed by Giemsa staining of blood smears with positive cases showing two pairs of small pear-shaped bi-lobed parasites [17]. In contrast, the presence of *B. bigemina* in a blood smear may not necessarily indicate clinical babesiosis, as symptoms can be due to resurgence of a chronic infection. In Kenya, diagnosis of redwater is confirmed by observation of the characteristic red urine, whereas microscopic examination of blood smears is seldom performed. In national diagnostic and research laboratories, detection of circulating *B. bigemina* antibodies is used for disease surveillance but this has its own shortcomings [21, 22, 35]. Molecular PCR techniques have been used to detect and differentiate *Babesia* parasites with high sensitivity and specificity [36–38]. Based on PCR amplification and sequencing of the *B. bovis* spherical body protein-4 (SBP-4) gene, Moumouni et al. [27] previously reported a *B. bovis* prevalence of 12.3% and 23.7% in Kajiado and Machakos Counties in Kenya, respectively. Using the reverse line blot (RLB) assay targeting the V4 hypervariable region of *Babesia* rRNA, Njiiri et al. [26] reported a *B. bovis* prevalence of 2.2% in Busia County, Western Kenya. In this study we used a well-validated TaqMan probe multiplex assay based on *B. bovis* 18S [28], *B. bovis* cytochrome *b* and *B. bigemina* cytochrome *b* [29] that can detect these species and differentiate *B. bovis* from *B. bigemina*. High specificities and efficiencies of > 96% were observed with these primer and probe sets (Fig. 2), confirming the efficiency and usefulness of these assays in detecting and discriminating *B. bovis* infections. In our study, a total of 61 (12.1%) and 44 (8.7%) animals were found to have single *B. bovis* and *B. bigemina* infections, respectively, while 27 (5.1%) animals were found to have *B. bovis* and *B. bigemina* co-infections. The presence of co-infections indicates the co-occurrence of the two *Boophilus* spp., *Rhipicephalus decoloratus*, which transmits *B. bigemina* and is endemic in Kenya, and *R. microplus*, the known vector for *B. bovis* that also transmits *B. bigemina*. Further studies on tick infections, species densities and their distribution are required to define the contribution of each species to the epidemiology of redwater in the country. The difference in sensitivities between the *B. bovis* 18S and cytochrome *b* primers may be due to differences in abundance of the two genes. The *B. bovis* genome has been shown to contain three rRNA operons [39]. Although information on the number of mitochondria in *Babesia* parasites is lacking, some apicomplexan parasites such as *Toxoplasma gondii* and *Plasmodium falciparum* have been reported to have only a single mitochondrion per parasite [40]. The mitochondrial DNA of *P. falciparum* comprises approximately 20 copies of a 6-kbp linear genome per cell [41, 42] encoding three protein coding genes including cytochrome *b* [39]. Therefore, the quantities detected by different genes could reflect the gene copies per individual organism as well as level of parasitaemia, which was not determined in this study. More *B. bovis*-positive samples were detected with the cytochrome *b* primers compared to the 18S indicating the higher sensitivity obtained by using this gene compared to the nuclear-encoded 18S rRNA gene. Therefore, based on the data obtained in this study and theoretical predictions, the *B. bovis* cytochrome *b* primers would be the best target for a routine field diagnostic assay.

Animals that recover from babesiosis become carriers of babesia parasites for life and can develop the disease again if they undergo physiological stresses such as nutritional restriction or co-infection with another infection. Babesiosis is therefore a costly chronic animal disease in endemic areas because of frequent resurgence, especially if the animals are experiencing stress [2]. Factors such as animal breed, type of agro-ecological zone (AEZ) and livestock production systems are important risk factors associated with babesiosis [22]. Most farmers in Kwale County, which has an estimated 190,988 zebu cattle and 5475 dairy crosses [43], practice an open grazing system, which has previously been shown to be significantly associated with high prevalence of *B. bigemina* infections in Murang’a County in Kenya [22]. Kwale County, which is a gateway to mainland Kenya for livestock purchased from the Tanzanian border market, could be acting as a focal source of *B. bovis* infections to the rest of the country.

Ticks and tick-borne diseases are the biggest threat to sustainable cattle production in Kenya [2], which has close to 18 million cattle [44]. Although this study reports high prevalence of *B. bovis* in three sub-counties in Kwale, the situation in the rest of the country is unknown. The emergence of *B. bovis* infections in Kenya threatens the already fragile livestock sector, plagued by other tick-borne diseases such as East Coast fever, and the introduction of susceptible taurine cattle breeds [45]. Therefore, the demonstration of a high prevalence of *B. bovis* revealed its establishment and local transmission. It is recommended that molecular diagnostics including the real-time PCRs used in this study be added to routine surveillance to detect and differentiate the two *Babesia* parasites with the aim of elucidating the current status of bovine babesiosis in the country. Such efforts will underpin the deployment of strategic disease control strategies for Kenya and its neighbours.

## Conclusion

In conclusion, we found a relatively high number of *B. bovis* and *B. bigemina* infections in asymptomatic cattle from Kwale County in Kenya with potential to cause disease outbreaks in susceptible animal populations. The *Babesia* multiplex real-time PCR used in this study is specific and can detect and differentiate the two *Babesia* parasites. The *B. bovis* cytochrome *b* primers would be the best target for a routine field diagnostic assay. Active surveillance to monitor the spread of *B. bovis* babesiosis across the country is recommended.

## Supplementary Information

**Additional file 1: Table S1.** Details of real-time PCR and SBP-4 nested PCR primers used in detection of *Babesia* parasites.

**Additional file 2: Table S2.** List of cattle blood samples screened for *Babesia bovis* and *B. bigemina* and the corresponding Cq scores obtained with the real-time PCR probes used in the current study.

## Data Availability

The nucleotide dataset(s) supporting the conclusions of this article have been deposited in GenBank repository (http://www.ncbi.nlm.nih.gov/genbank/). The 24 field and three reference *B. bovis* SBP-4 sequences are under the accession numbers ON012652-ON012678. All other data sets supporting the conclusions of this article have been presented.

## Abbreviations

EDTA: Ethylenediaminetetraacetic acid
Cq: Cycle quantification
DNA: Deoxyribonucleic acid
dNTPs: Deoxyribonucleotide triphosphates
ILRI: International Livestock Research Institute
rDNA: Ribosomal deoxyribonucleic acid
qPCR: Quantitative PCR
TFC: Tick fever centre
