# Supplementary material for: Molecular survey of Babesia parasites in Kenya: first detailed report on occurrence of Babesia bovis in cattle
Source: Parasit Vectors. 2022 May 7;15:161. doi: 10.1186/s13071-022-05279-7 (PMC9077973; doi:10.1186/s13071-022-05279-7)
Supplement: Supplementary file 1 — Additional file 1: Table S1. Details of real-time PCR and SBP-4 nested PCR primers used in detection of Babesia parasites. [file 13071_2022_5279_MOESM1_ESM.docx]

| **Target gene** | **Primer/probe name** | **Nucleotide sequence** | **Product size** | **Reference** |
| --- | --- | --- | --- | --- |
| **Real-time PCR:**  *Babesia bovis* 18S | Forward: S0934_BoF_18S | AGCAGGTTTCGCCTGTATAATG | 154 | [28] |
|  | Reverse: S0933_BoR_18S | AGTCGTGCGTCATCGACAAA |  |  |
|  | Probe:  S0935_BoP_Kim | FAM-CCTTGTATGACCCTGTCGTACCGTTGG-BHQ1 |  |  |
| **Real-time PCR:**  *Babesia bovis* Cytochrome *b (cytb)* | Forward: S0936_bovisF160_cytb | ATATGTTTGCATTTGCTG | 90 | [29] |
|  | Reverse: S0937_bovisR249_cytb | CTCCAAACCAATATGAAAG |  |  |
|  | Probe: S0938_bovisPb_cytb | JOE- CAAACCATAAAGTCATCGGTATATCCTAC-BHQ1 |  |  |
| **Real-time PCR:**  *Babesia bigemina*  Cytochrome *b (cytb)* | Forward:  bigemF295 | GGTCTATTTGGTGGAGTT | 146 | [29] |
|  | Reverse: bigemR413 | ACAAGACCAAATGCAATT |  |  |
|  | Probe: bigemPb | TAMRA-CAATTGTTCTTGGAGCAGCT- BHQ1 |  |  |
| **Conventional PCR:**  *Babesia bovis* SBP-4 | Outer PCR: Forward | AGTTGTTGGAGGAGGCTAAT | 907 | [30] |
|  | Outer PCR: Reverse | TCCTTCTCGGCGTCCTTTTC |  |  |
|  | Nested PCR: Forward | GAAATCCCTGTTCCAGAG | 503 |  |
|  | Reverse | TCGTTGATAACACTGCAA |  |  |
