# Supplementary material for: Molecular survey of Babesia parasites in Kenya: first detailed report on occurrence of Babesia bovis in cattle
Source: Parasit Vectors. 2022 May 7;15:161. doi: 10.1186/s13071-022-05279-7 (PMC9077973; doi:10.1186/s13071-022-05279-7)
Supplement: Supplementary file 2 — Additional file 2: Table S2. List of cattle blood samples screened for Babesia bovis and B. bigemina and the corresponding Cq scores obtained with the real-time PCR probes used in the current study. [file 13071_2022_5279_MOESM2_ESM.docx]

| **Serial Number** | **Voucher Number** | **Sampling site** | **Sub-county** | **Cycle quantification (Cq) scores ^a^** | | |
| --- | --- | --- | --- | --- | --- | --- |
|  |  |  |  | ***Babesia bovis* 18S** | ***Babesia bovis* Cytochrome *b*** | ***Babesia bigemina***  **Cytochrome *b*** |
| 1 | 1 | Matuga | Matuga | 18.7 | - | 36.7 |
| 2 | 2 | Matuga | Matuga | - | - | 31.9 |
| 3 | 3 | Matuga | Matuga | - | 37.1 | 32.3 |
| 4 | 4 | Matuga | Matuga | 38.2 | 32.2 | 37.9 |
| 5 | 6 | Matuga | Matuga | - | 37.6 | 27.5 |
| 6 | 9 | Matuga | Matuga | - | 40.3 | - |
| 7 | 12 | Matuga | Matuga | - | - | 28.4 |
| 8 | 16 | Miaji Farm | Matuga | - | 39.1 | 35.3 |
| 9 | 22 | Miaji Farm | Matuga | - | 39.8 | 35.9 |
| 10 | 23 | Miaji Farm | Matuga | - | 40.9 | - |
| 11 | 24 | Miaji Farm | Matuga | - | - | 34.6 |
| 12 | 25 | Miaji Farm | Matuga | - | - | 34.4 |
| 13 | 26 | Miaji Farm | Matuga | - | - | 34.4 |
| 14 | 29 | Miaji Farm | Matuga | - | - | 38.0 |
| 15 | 30 | Miaji Farm | Matuga | - | - | 41.3 |
| 16 | 32 | Miaji Farm | Matuga | 39.8 | 31.1 | - |
| 17 | 33 | Miaji Farm | Matuga | - | - | 32.8 |
| 18 | 35 | Miaji Farm | Matuga | - | - | 32.6 |
| 19 | 36 | Miaji Farm | Matuga | - | - | 31.7 |
| 20 | 44 | Miaji Farm | Matuga | - | - | 33.0 |
| 21 | 45 | Miaji Farm | Matuga | - | - | 36.3 |
| 22 | 46 | Miaji Farm | Matuga | - | - | 29.2 |
| 23 | 48 | Miaji Farm | Matuga | - | - | 37.0 |
| 24 | 50 | Miaji Farm | Matuga | - | - | 31.8 |
| 25 | 51 | Miaji Farm | Matuga | - | - | 34.5 |
| 26 | 54 | Miaji Farm | Matuga | - | - | 36.0 |
| 27 | 56 | Kipabane | Matuga | - | 35.8 | 35.2 |
| 28 | 60 | Kipabane | Matuga | 40.4 | 32.7 | 33.3 |
| 29 | 64 | Kipabane | Matuga | - | - | 35.0 |
| 30 | 75 | Kipabane | Matuga | - | 41.0 | - |
| 31 | 80 | Kipabane | Matuga | - | 36.2 | - |
| 32 | 87 | Kipabane | Matuga | - | 40.8 | - |
| 33 | 88 | Kipabane | Matuga | - | 40.8 | - |
| 34 | 95 | Kipabane | Matuga | 38.4 | 32.1 | - |
| 35 | 96 | Kipabane | Matuga | 41.5 | 35.5 | 30.5 |
| 36 | 102 | Tangini | Msambweni | - | - | 28.9 |
| 37 | 104 | Tangini | Msambweni | - | - | 30.5 |
| 38 | 106 | Tangini | Msambweni | - | - | 38.2 |
| 39 | 108 | Tangini | Msambweni | - | - | 26.9 |
| 40 | 113 | Tangini | Msambweni | 40.3 | 35.9 | 28.9 |
| 41 | 120 | Tangini | Msambweni | 40.3 | 32.9 | - |
| 42 | 125 | Tangini | Msambweni | - | - | 30.1 |
| 43 | 140 | Tangini | Msambweni | - | - | 35.5 |
| 44 | 147 | Tangini | Msambweni | - | - | 35.3 |
| 45 | 151 | Tangini | Msambweni | 32.8 | - | - |
| 46 | 155 | Tangini | Msambweni | - | - | 33.6 |
| 47 | 156 | Tangini | Msambweni | - | - | 41.4 |
| 48 | 175 | Majiboni | Msambweni | 39.1 | 31.3 | - |
| 49 | 181 | Majiboni | Msambweni | - | 35.0 | - |
| 50 | 190 | Majiboni | Msambweni | - | 33.0 | - |
| 51 | 202 | Kichaka simba | Matuga | - | 38.0 | - |
| 52 | 207 | Kichaka simba | Matuga | - | 38.6 | - |
| 53 | 218 | Kichaka simba | Matuga | - | 38.2 | - |
| 54 | 221 | Kichaka simba | Matuga | - | 37.6 | - |
| 55 | 222 | Kichaka simba | Matuga | - | 38.5 | - |
| 56 | 228 | Kichaka simba | Matuga | - | 38.0 | - |
| 57 | 229 | Kichaka simba | Matuga | - | 37.5 | - |
| 58 | 231 | Kichaka simba | Matuga | - | 38.0 | - |
| 59 | 241 | Kichaka simba | Matuga | - | 38.8 | - |
| 60 | 243 | Kichaka simba | Matuga | - | 38.3 | - |
| 61 | 247 | Kichaka simba | Matuga | - | 38.0 | - |
| 62 | 249 | Kichaka simba | Matuga | - | 38.5 | - |
| 63 | 262 | Kichaka simba | Matuga | - | 38.5 | - |
| 64 | 269 | Kichaka simba | Matuga | - | 37.9 | - |
| 65 | 273 | Kichaka simba | Matuga | - | 37.3 | - |
| 66 | 275 | Kichaka simba | Matuga | - | 38.1 | - |
| 67 | 278 | Kichaka simba | Matuga | - | 38.7 | - |
| 68 | 313 | Ukunda | Msambweni | - | - | 33.4 |
| 69 | 322 | Ukunda | Msambweni | - | - | 30.8 |
| 70 | 323 | Ukunda | Msambweni | 37.4 | 33.4 | 33.7 |
| 71 | 341 | Shimoni Kidimu | Lunga Lunga | 36.4 | 32.5 | 33.9 |
| 72 | 342 | Shimoni Kidimu | Lunga Lunga | - | 32.6 | - |
| 73 | 343 | Shimoni Kidimu | Lunga Lunga | - | - | 35.2 |
| 74 | 344 | Shimoni Kidimu | Lunga Lunga | - | 31.7 | 32.3 |
| 75 | 345 | Shimoni Kidimu | Lunga Lunga | - | - | - |
| 76 | 346 | Shimoni Kidimu | Lunga Lunga | - | 32.4 | - |
| 77 | 349 | Shimoni Kidimu | Lunga Lunga | - | - | 36.4 |
| 78 | 350 | Shimoni Kidimu | Lunga Lunga | - | - | 34.5 |
| 79 | 351 | Shimoni Kidimu | Lunga Lunga | - | 36.3 | 36.4 |
| 80 | 352 | Shimoni Kidimu | Lunga Lunga | - | - | 38.0 |
| 81 | 354 | Shimoni Kidimu | Lunga Lunga | 34.6 | - | - |
| 82 | 355 | Shimoni Kidimu | Lunga Lunga | - | 31.8 | 37.7 |
| 83 | 356 | Shimoni Kidimu | Lunga Lunga | 36.4 | 32.8 | 32.1 |
| 84 | 357 | Shimoni Kidimu | Lunga Lunga | 36.7 | 32.8 | 33.6 |
| 85 | 371 | Shimoni Kidimu | Lunga Lunga | - | - | - |
| 86 | 380 | Kiwambale | Lunga Lunga | 24.4 | 32.9 | - |
| 87 | 384 | Kiwambale | Lunga Lunga | 36.2 | - | - |
| 88 | 390 | Kiwambale | Lunga Lunga | 38.3 | 34.4 | 35.2 |
| 89 | 402 | Kiwambale | Lunga Lunga | - | - | 30.2 |
| 90 | 409 | Shimoni | Lunga Lunga | - | 32.9 | - |
| 91 | 419 | Shimoni | Lunga Lunga | 38.5 | 32.2 | - |
| 92 | 421 | Shimoni | Lunga Lunga | 36.6 | 32.8 | 35.4 |
| 93 | 423 | Shimoni | Lunga Lunga | - | - | 36.2 |
| 94 | 427 | Shimoni | Lunga Lunga | 36.4 | 32.1 | - |
| 95 | 430 | Shimoni | Lunga Lunga | 35.0 | 29.9 | - |
| 96 | 437 | Shimoni | Lunga Lunga | - | - | 32.3 |
| 97 | 440 | Shimoni | Lunga Lunga | 43.1 | 33.7 | - |
| 98 | 441 | Vanga | Lunga Lunga | 13.0 | - | - |
| 99 | 446 | Vanga | Lunga Lunga | - | - | 27.9 |
| 100 | 447 | Vanga | Lunga Lunga | - | - | 33.8 |
| 101 | 448 | Vanga | Lunga Lunga | 30.8 | - | - |
| 102 | 449 | Vanga | Lunga Lunga | - | - | 30.9 |
| 103 | 450 | Vanga | Lunga Lunga | - | 33.0 | 35.7 |
| 104 | 451 | Vanga | Lunga Lunga | 37.1 | 33.6 | - |
| 105 | 452 | Vanga | Lunga Lunga | - | 35.6 | - |
| 106 | 456 | Vanga | Lunga Lunga | - | 35.8 | - |
| 107 | 458 | Vanga | Lunga Lunga | - | 38.3 | - |
| 108 | 460 | Vanga | Lunga Lunga | 39.2 | 35.4 | - |
| 109 | 463 | Vanga | Lunga Lunga | 38.6 | 34.4 | - |
| 110 | 468 | Vanga | Lunga Lunga | 44.7 | 33.6 | - |
| 111 | 470 | Vanga | Lunga Lunga | 37.2 | 32.2 | 34.6 |
| 112 | 472 | Vanga | Lunga Lunga | - | 34.7 | - |
| 113 | 474 | Vanga | Lunga Lunga | - | 37.2 | - |
| 114 | 475 | Vanga | Lunga Lunga | 40.2 | 33.3 | - |
| 115 | 476 | Vanga | Lunga Lunga | - | - | 29.7 |
| 116 | 477 | Vanga | Lunga Lunga | - | 34.2 | 31.9 |
| 117 | 478 | Vanga | Lunga Lunga | - | - | 21.0 |
| 118 | 479 | Vanga | Lunga Lunga | - | 35.9 | - |
| 119 | 487 | Vanga | Lunga Lunga | - | - | 25.6 |
| 120 | 488 | Vanga | Lunga Lunga | 37.0 | 32.3 | 32.5 |
| 121 | 489 | Vanga | Lunga Lunga | 38.9 | 34.2 | - |
| 122 | 490 | Vanga | Lunga Lunga | 37.8 | 33.5 | 35.6 |
| 123 | 492 | Vanga | Lunga Lunga | - | 39.1 | 33.8 |
| 124 | 493 | Vanga | Lunga Lunga | - | - | 22.3 |
| 125 | 495 | Vanga | Lunga Lunga | 35.9 | 31.2 | 33.589 |
| 126 | 496 | Vanga | Lunga Lunga | 42.9 | 35.2 | - |
| 127 | 497 | Vanga | Lunga Lunga | 36.5 | 31.3 | 28.1 |
| 128 | 498 | Vanga | Lunga Lunga | - | 33.6 | - |
| 129 | 499 | Vanga | Lunga Lunga | 38.8 | 30.1 | - |
| 130 | 500 | Vanga | Lunga Lunga | 37.8 | 34.4 | - |
| 131 | 501 | Vanga | Lunga Lunga | - | 41.2 | - |
|  |  |  | **Mean Cq** | **36.6** | **35.4** | **33.2** |

^a^ Values were rounded off to the nearest one decimal point
